# Comparative effectiveness of neoadjuvant therapy combined with stent placement versus stent alone in obstructive colorectal cancer: a multicenter retrospective analysis

**DOI:** 10.1186/s12885-026-16728-2

**Published:** 2026-09-24

**Authors:** Zhuo Han, Bo Zhang, Shiqi Liu, Tao Wu, Qing Qiao, Xianli He, Jiagang Han, Nan Wang

**Affiliations:** 1 Department of General Surgery, Gastrointestinal Surgery Center, Tangdu Hospital, The Fourth Military Medical University, Xi’an, China; 2 Department of General Surgery, Beijing Chaoyang Hospital, Capital Medical University, Beijing, China

**Keywords:** Colorectal cancer, Bowel obstruction, Neoadjuvant therapy, SEMS, Survival outcome

## Abstract

**Objective:**

The aim of this study was to evaluate the impact of self-expandable metal stents (SEMS) and neoadjuvant therapy (NAT) for obstructive colorectal cancer (CRC).

**Methods:**

This multicenter, retrospective study analyzed 154 patients with obstructive CRC from three centers between 2011 and 2025. Patients were allocated to either a NAT combined with SEMS group (*n* = 93) or a SEMS-alone group (*n* = 61). Short-term procedural outcomes, pathological tumor response, long-term survival, and the impact of the time interval from stenting to surgery were evaluated between groups and across two time periods (2011–2020 vs. 2021–2025).

**Results:**

Baseline characteristics were largely comparable between groups. The NAT group demonstrated significantly lower intraoperative stoma rates (8.6% vs. 29.0%, *P* = 0.031) and postoperative complication rates (8.6% vs. 29.0%, *P* = 0.031) in the 2011–2020, advantages that persisted in the 2021–2025. Pathological assessment revealed a superior tumor response in the 2021–2025, with 20.7% achieving TRG 0–1 and significantly higher rates of T and N downstaging (39.7% and 60.3%) compared to the 2011–2020. Although survival benefits were not significant in the 2011–2020 period, NAT may be associated with a significant improvement in overall survival (OS) in the 2021–2025 cohort (*P* = 0.013). Analysis of surgical timing identified an optimal interval of 14–21 days related to OS for the SEMS group, whereas for NAT patients, an interval exceeding 90 days was associated with worse disease-free survival (DFS).

**Conclusion:**

SEMS-NAT for obstructive CRC may is an important factor for improved surgical quality and OS benefit in 2021–2025. This study provides crucial evidence for optimizing treatment strategy by recommending a surgery interval.

**Supplementary Information:**

The online version contains supplementary material available at https://doi.org/10.1186/s12885-026-16728-2.

## Introduction

Colorectal cancer (CRC) continues to represent a major global health concern. Recent epidemiological studies have identified CRC as the third most frequently diagnosed cancer and the second leading cause of cancer-related mortality worldwide, thereby contributing significantly to morbidity and healthcare system burdens [1]. A critical complication among CRC patients is malignant large bowel obstruction, which affects approximately 15–29% of cases and frequently requires emergency surgical intervention [2]. Previous clinical feature analyses have shown that, compared to non-obstructive CRC, patients with obstructive CRC more frequently have tumors located in the left colon, larger tumor sizes, a higher proportion of moderately to poorly differentiated tumors, and are diagnosed at more advanced tumor stages, with an increased tendency for peritoneal metastasis [3, 4]. Additionally, multiple studies have demonstrated that obstruction is closely associated with inflammatory markers [5], with higher inflammatory burden index (IBI) scores significantly correlating with poorer disease-free survival (DFS) and overall survival (OS) [6]. Concurrently, obstructive CRC patients exhibit worsened nutritional status (hypoproteinemia), which further impairs treatment tolerance and postoperative recovery [7]. Recent studies have identified unique molecular and pathological characteristics of obstructive CRC, including alterations in extracellular matrix components, increased matrix stiffness, activation of cancer-associated fibroblasts (CAFs), and positive feedback signaling loop between CAFs and sympathetic nerves that promotes tumor cell aggregation and migration. Additionally, the immune microenvironment of obstructive CRC exhibits a significantly higher density of CD8 + T cells compared to non-obstructive CRC, which paradoxically correlates with a poorer prognosis [8, 9]. Regarding treatment response, no comparative studies have yet evaluated the neoadjuvant efficacy between obstructive CRC and non-obstructive CRC patients, however, the remission observed in obstructed patients following neoadjuvant therapy suggests that this regimen is feasible [10, 11]. Most studies indicate that the 5-year OS and DFS rates for obstructive CRC patients are significantly worse than those for non-obstructive CRC patients, with obstruction status remaining an independent risk factor for OS [3, 4]. Based on the characteristics described above, the treatment of obstructive CRC has consistently been a primary focus of research. Emergency surgery (ES) for obstructive CRC is associated with increased risks, including perioperative mortality rates as high as 10–15%, elevated morbidity such as anastomotic leakage and wound infections, and a greater probability of permanent stoma formation. These factors collectively contribute to poor overall survival outcomes and reduced quality of life [12].

Since their introduction in the 1990s, self-expandable metal stents (SEMS) have become a crucial minimally invasive alternative to ES, providing immediate decompression of obstruction, symptom relief, and serving as a bridge to elective surgery [13]. The placement of SEMS has demonstrated notable short-term advantages, including lower early complication rates (13.6% vs. 25.5% in emergency surgery), reduced mortality rates (3.9% vs. 9.4%), and decreased stoma formation rates (14.3% vs. 51.4%), as evidenced by meta-analyses and clinical studies [14, 15]. However, accumulating evidence suggests that SEMS may not confer significant advantages in long-term oncological outcomes when compared to direct emergency surgery or elective surgery without stenting [16]. A prospective study conducted by K. J. Gorissen et al. reported that among patients < 75 years, the local recurrence rate was significantly higher in the SEMS group compared to the ES group (32% vs. 8%, *P* = 0.038), potentially attributable to factors such as neural invasion or microperforations incurred during stent deployment [17].

These findings raise concerns that SEMS, while effective for acute decompression, may inadvertently promote tumor progression or limit the potential for radical resection, thereby potentially diminishing their initial benefits. The prevailing consensus indicates that SEMS alone does not adequately address the fundamental challenge of improving long-term prognosis in patients with obstructive CRC [18, 19]. This limitation highlights the necessity of neoadjuvant therapy (NAT) to enhance the oncological safety and efficacy of stent-based management. To address the shortcomings of SEMS in terms of long-term outcomes, the integration of NAT (comprising chemotherapy and/or radiotherapy) has been proposed as a strategic approach to augment the therapeutic benefits of stent placement in obstructive CRC patients [20, 21]. The theoretical rationale for this combined approach is multifaceted: NAT administered following SEMS decompression may reduce peri-tumoral inflammation and bowel edema, facilitate tumor downstaging, increase the likelihood of achieving R0 resection margins, and potentially eradicate micrometastases [22]. However, current research is limited by small sample sizes, methodological heterogeneity, and a paucity of high-level evidence, resulting in inconclusive findings regarding the superiority of this combined modality. Therefore, there is an urgent need for well-designed studies to validate the feasibility, safety, and oncological benefits of incorporating NAT into the SEMS treatment pathway, with particular emphasis on optimizing timing and patient selection to maximize therapeutic outcomes.

This retrospective analysis seeks to address existing research gaps by comprehensively evaluating the short-term efficacy and long-term prognostic outcomes of NAT combined with SEMS versus SEMS alone in patients with obstructive colorectal cancer. Additionally, the study aims to determine the optimal timing for surgical intervention following stent placement, a critical yet insufficiently explored factor affecting both operative success and patient survival.

## Methods

### Study design and patients

This multi-center retrospective cohort study included patients with obstructive CRC who received SEMS placement followed by curative surgical resection. The recruited patients were from Tangdu Hospital, Chaoyang Hospital, and Xijing Hospital, with 71, 59, and 24 patients enrolled from each center, respectively. The study was conducted from January 2011 to December 2025. All three centers use a unified electronic case report form for data collection, including follow-up data, and the main center uniformly conducts the logical validation of the data. The research adhered to the Declaration of Helsinki and received approval from the Institutional Review Boards (IRBs) or Ethics Committees of all three hospitals. Detailed inclusion and exclusion criteria are provided in the supplementary materials.

### Treatment measure

Patients were divided into two groups according to their post-stent management.

SEMS-NAT Group: patients received SEMS to alleviate obstruction, followed by neoadjuvant therapy individualized to their clinical condition, and subsequently underwent surgery. Following successful stent placement and resolution of the obstruction, multidisciplinary team (MDT) at each center assess the appropriateness of administering neoadjuvant therapy based on standardized criteria. Indications for NAT include: (1) locally advanced disease, as indicated by imaging findings consistent with cT_3−4_N+, (2) molecular subtyping, with immunotherapy prioritized for patients exhibiting deficient mismatch repair (dMMR) or microsatellite instability-high (MSI-H) status, (3) resectability assessment, particularly for patients requiring an extended interval between stent placement and surgery to facilitate tumor downstaging, and (4) performance status, specifically an Eastern Cooperative Oncology Group (ECOG) performance status of 0–1, indicating the patient’s capacity to tolerate systemic therapy. The neoadjuvant treatment regimens included 54 patients treated with XELOX, 21 with FOLFOX, 4 with XELOX plus cetuximab, 7 with XELOX plus immunotherapy, 2 with FOLFOX plus cetuximab, and 5 patients had unknown regimens. In clinical practice, neoadjuvant therapy combined with cetuximab is limited to patients with RAS/BRAF wild-type tumors located in the left colon or rectum, whereas combined immunotherapy is limited to patients confirmed to have MSI-H/dMMR status through polymerase chain reaction (PCR) or immunohistochemistry (IHC) testing.

SEMS group: consisted of patients who underwent SEMS placement for obstruction relief and proceeded directly to surgery without receiving neoadjuvant therapy, the timing of surgery was determined based on clinical considerations and scheduling factors.

This study utilized standardized criteria to assess tumor resectability, encompassing the following dimensions: (1) anatomical factors, including evaluation of circumferential resection margins, presence of extramural vascular invasion, depth of tumor infiltration, and involvement of adjacent organs as determined by imaging, (2) oncological considerations, prioritizing the achievement of R0 resection, with inclusion of patients presenting with oligometastases when curative treatment was deemed feasible, (3) functional and physiological status, involving assessment of ECOG performance status, nutritional condition, and vital organ function, with exclusion of patients deemed unfit for surgery. All decisions were made by consensus through MDT discussions at each participating center.

### Observation indices

The collected data encompassed patient demographics, perioperative outcomes, and surgical parameters. Key variables included age, sex, body mass index (BMI), chronic comorbidities (including hypertension, diabetes mellitus, and coronary heart disease), American Society of Anesthesiologists (ASA) score, preoperative hemoglobin and albumin levels, tumor location, clinical stage, details of neoadjuvant therapy, stent-to-surgery interval, and stent-related complications. Surgical parameters comprised the surgical approach (laparoscopic or open), operative duration, intraoperative blood loss, stoma rate, lymph node assessment, tumor characteristics, pathological stage, Tumor Regression Grade (TRG, AJCC), postoperative complications, and length of hospital stay.

### Primary endpoints

The study primarily investigated OS and DFS in patients receiving SEMS-NAT and SEMS groups. OS was defined as the duration from surgery to death from any cause, while DFS was defined as the interval from initial radical resection to either the first recurrence or the last follow-up. Furthermore, the study evaluated the effect of the interval between stent placement and surgery on patient prognosis, aiming to determine the optimal timing for surgical intervention in each group.

### Statistical analysis

To address potential biases from the study period (2011–2025), a stratified analysis was performed across two distinct time intervals: 2011–2020 and 2021–2025. According to the previous literature reports and guideline recommendations, stent-to-surgery intervals were categorized as ≤ 14 days, 14–21 days, and ≥ 21 days [23, 24]. While neoadjuvant therapy intervals were divided into < 90 days and ≥ 90 days based on the clinical practice.

Statistical analyses were conducted using SPSS (version 27.0) and R (version 4.3.2). Continuous variables were presented as mean ± standard deviation (SD) or median with interquartile range (IQR). The Student’s t-test or Mann–Whitney U test were adopted to analyzed depending on the data distribution. Categorical variables were expressed as frequencies (%) and compared using the Chi-square test or Fisher’s exact test. Survival outcomes, including OS and DFS, were analyzed through Kaplan-Meier (KM) curve and cox regression. A two-sided *P* < 0.05 was considered statistically significant for all analysis.

## Results

### Baseline characteristics of patients

**Fig. 1:**
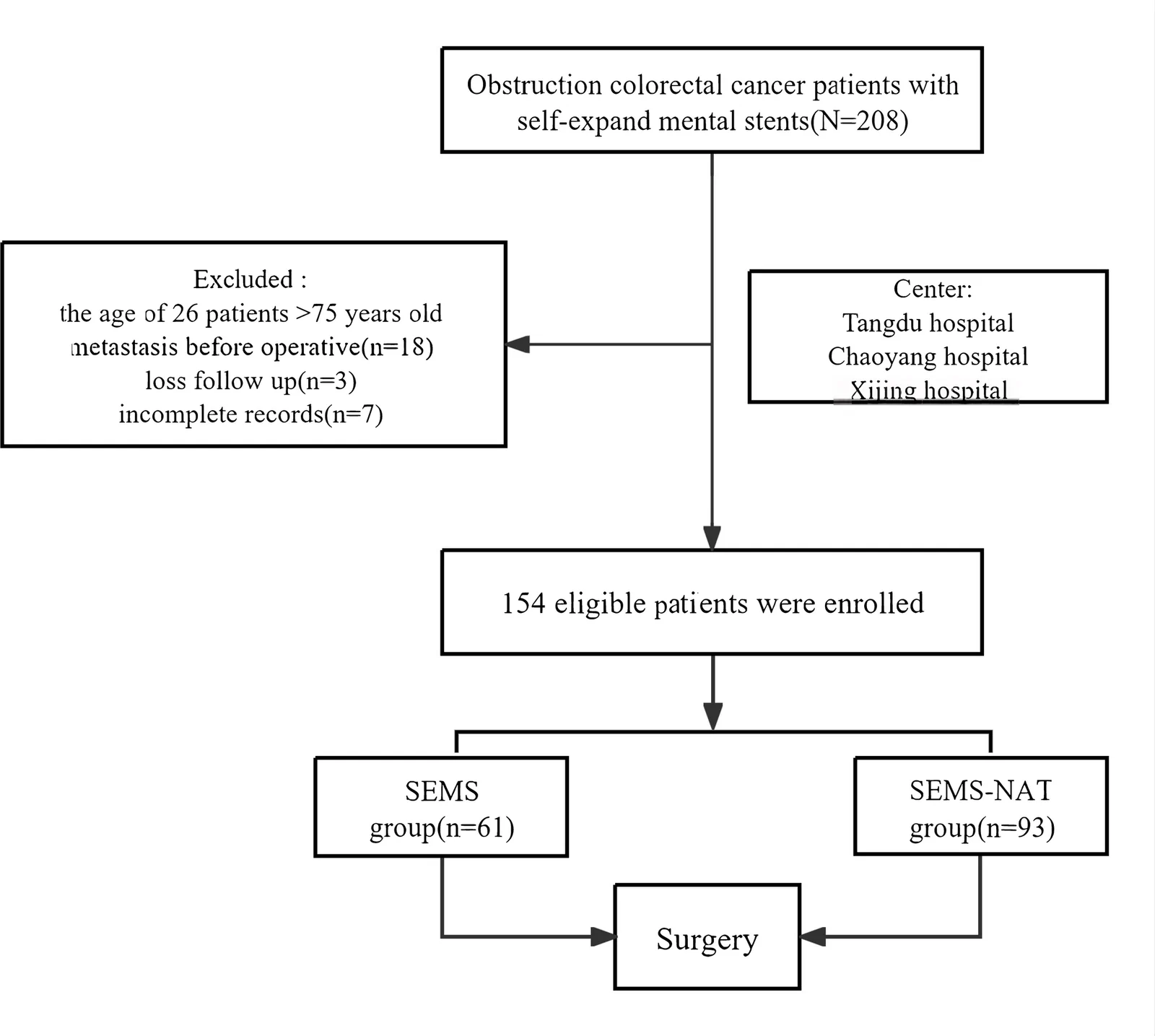
The screening process of included patients in this study

The study included 154 patients with obstructive colorectal cancer, as illustrated in Fig. 1. Among these, 93 patients were assigned to the SEMS-NAT group, while 61 were in the SEMS group. From 2011 to 2020, 31 patients were in the SEMS group and 35 in the SEMS-NAT group, with no significant baseline differences were observed except for preoperative hemoglobin levels (118.32 ± 16.81 g/L vs. 103.71 ± 19.07 g/L, *P* = 0.002). From 2021 to 2025, 30 patients were in the SEMS group and 58 in the SEMS-NAT group, with no significant baseline differences identified. During the 2011–2020 period, the SEMS-NAT group experienced 2 cases of re-obstruction (5.7%) and 1 perforation (2.9%), whereas the SEMS group reported 1 case of re-obstruction (3.2%). In the 2021–2025 period, the SEMS group had 1 perforation (3.3%), and the SEMS-NAT group had 4 re-obstructions (6.9%), along with 1 stent migration and 1 perforation (each 1.7%). No significant differences in stent-related complications were observed between the SEMS-NAT and SEMS groups during either period. Regarding treatment, from 2011 to 2020, 97.1% of patients in the SEMS-NAT group received chemotherapy alone, with a median of 2 cycles. From 2021 to 2025, 22.4% of patients received chemotherapy combined with targeted or immunotherapy, and the median number of cycles increased to 3. Detailed information is provided in Table 1.

**Table 1:** Comparison of baseline characteristics between SEMS-NAT group and SEMS group

Characteristics	2011–2020				2021–2025			
	SEMS group(*N* = 31)	SEMS-NAT group(*N* = 35)	Z/t/χ^2^	*P*	SEMS group(*N* = 30)	SEMS-NAT group(*N* = 58)	Z/t/χ^2^	*P*
Age, M(range)	61.0(29.0,75.0)	64.0(28.0,75.0)	-0.785	0.433	59.0(21.0,75.0)	58.0(26.0,74.0)	-1.519	0.129
Gender, *n* (%)			0.045	0.833			0.241	0.624
male	22(71.0)	24(68.6)			17(56.7)	36(62.1)		
female	9(29.0)	11(31.4)			13(43.3)	22(37.9)		
BMI,$\:\overline{\boldsymbol{x}}\pm\:\boldsymbol{s}$	22.06±2.97	22.46±3.45	-0.500	0.619	21.99±2.58	22.28±3.15	-0.433	0.666
Preoperative hemoglobin	118.32±16.81	103.71±19.07	3.282	**0.002**	118.83±19.57	112.83±19.08	1.388	0.169
Preoperative albumin	33.49±8.28	33.56±6.17	-0.037	0.970	35.21±5.01	37.79±7.60	-1.676	0.097
Chronic disease history, *n* (%)			0.122	0.727			0.344	0.558
yes	11(35.5)	11(31.4)			12(40.0)	27(46.6)		
no	20(64.5)	24(68.6)			18(60.0)	31(53.4)		
Tumor location, *n* (%)			5.821	0.059			4.149	0.246
sigmoid colon	27(87.1)	29(82.9)			21(70.0)	39(67.2)		
transverse colon	2(6.5)	0(0.0)			2(6.7)	6(10.3)		
right hemicolon	1(3.2)	0(0.0)			4(13.3)	2(3.4)		
rectum	1(3.2)	6(17.1)			3(10.0)	11(19.0)		
ASA score, *n* (%)			1.706	0.192			0.365	0.546
≤2	27(87.1)	26(74.3)			26(86.7)	54(93.1)		
≥3	4(12.9)	9(25.7)			4(13.3)	4(6.9)		
Clinical T stage, *n* (%)			0.398	0.528			3.659	0.161
T3	6(19.4)	3(8.6)			7(23.3)	10(17.2)		
T4	20(64.5)	25(71.4)			20(66.7)	43(74.1)		
NA	5(16.1)	7(20.0)			3(10.0)	5(8.6)		
Clinical N stage, *n* (%)			-	> 0.999			3.582	0.167
N0	7(22.6)	5(14.3)			7(23.3)	12(20.7)		
N+	22(71.0)	27(77.1)			22(73.3)	45(77.6)		
NA	2(6.5)	3(8.6)			1(3.3)	1(1.7)		
Interval time/d, M(Q1, Q3)	19.0(13.0,24.0)	74.0(56.0,86.0)	-5.875	**< 0.001**	15.5(10.8,19.5)	92.5(67.8,119.8)	-7.585	**< 0.001**
Complications related stent, *n* (%)								
perforation	0(0.0)	1(2.9)	-	> 0.999	1(3.3)	1(1.7)	-	> 0.999
reobstruction	1(3.2)	2(5.7)	-	> 0.999	0(0.0)	4(6.9)	0.869	0.351
fall off	0(0.0)	0(0.0)	-	-	0(0.0)	1(1.7)	-	> 0.999
Neoadjuvant therapy regimen, *n* (%)								
chemo therapy	-	34(97.1)			-	41(70.7)		
chemo-target therapy	-	0(0.0)			-	6(10.3)		
chemo-immune therapy	-	0(0.0)			-	7(12.1)		
other	-	1(2.9)			-	4(6.9)		
Neoadjuvant chemotherapy cycle, M(range)	-	2(1,4)				3(1,6)		

*SEMS* self-expandable metal stents, *NAT* neoadjuvant therapy, *BMI* body mass index, *ASA* American Society of Anesthesiologists

### Operation and pathology outcomes

According to Table 2, from 2011 to 2020, the SEMS-NAT group exhibited a higher rate of laparoscopic surgery compared to the SEMS group (85.7% vs. 67.7%, *P* = 0.082), although this difference was not statistically significant. No significant differences were observed in median operative time, intraoperative blood loss, length of hospital stay, or number of positive lymph nodes. The SEMS-NAT group demonstrated a greater number of lymph nodes harvested (26.0 vs. 21.0, *P* = 0.226) and a significantly lower intraoperative stoma rate (8.6% vs. 29.0%, *P* = 0.031), with all stomas being prophylactic. Additionally, the SEMS group experienced a higher rate of postoperative complications (29.0% vs. 8.6%, *P* = 0.031). From 2021 to 2025, the SEMS-NAT group exhibited lower rates of stoma formation (*P* = 0.180) and complications (*P* = 0.05) compared with SEMS group, however, these differences were not statistically significant. Notably, there was a significant reduction in the number of positive lymph nodes (*P* = 0.032).

**Table 2:** Operative and pathology outcome between SEMS-NAT group and SEMS group

Variables	2011–2020				2021–2025			
	SEMS group(*N* = 31)	SEMS-NAT group(*N* = 35)	Z/t/χ^2^	*P*	SEMS group(*N* = 30)	SEMS-NAT group(*N* = 58)	Z/t/χ^2^	*P*
Surgery type, *n* (%)			3.024	0.082			0.028	0.867
laparoscopic	21(67.7)	30(85.7)			26(86.7)	48(82.8)		
open	10(32.3)	5(14.3)			4(13.3)	10(17.2)		
Operative time, M (Q1, Q3)	200(180,240)	210(170,235)	-0.257	0.797	210(150,230)	205(180,240)	-0.696	0.486
Intraoperative blood loss, M (Q1, Q3)	100(50,150)	100(50,150)	-0.590	0.556	58(28,100)	50(40,100)	-0.314	0.754
Stoma, *n* (%)	9(29.0)	3(8.6)	4.626	**0.031**	4(13.3)	3(5.2)	1.799	0.180
Postoperative hospital, M(Range)	9(3.0,33.0)	9(4.0,33.0)	-0.383	0.702	7(4,15)	8(3,33)	-1.546	0.122
Lymphnodes removed, M (Q1, Q3)	21(17,32)	26(18,29)	-1.210	0.226	22(18,25)	24(19,31)	-1.710	0.087
Positive lymphnodes, M (Q1, Q3)	1(0,4)	1(0,2)	-0.479	0.632	1(0,3)	0(0,1)	-2.139	**0.032**
Tumor size, *n* (%)			2.344	0.126			0.352	0.553
<5 cm	20(64.5)	16(45.7)			13(43.3)	29(50.0)		
≥5 cm	11(35.5)	19(54.3)			17(56.7)	29(50.0)		
Degree of differentiation, *n* (%)			2.027	0.363			3.699	0.157
well-moderate	25(80.6)	31(88.6)			19(63.3)	41(70.7)		
poor	5(16.1)	2(5.7)			11(36.7)	13(22.4)		
other	1(3.2)	2(5.7)			0(0.0)	4(6.9)		
pT stage, *n* (%)			-	0.494			5.537	**0.019**
T_0−2_	0(0.0)	2(5.7)			0(0.0)	12(20.7)		
T_3−4_	31(100.0)	33(94.3)			30(100.0)	46(79.3)		
pN stage, *n* (%)			1.669	0.434			7.534	**0.023**
N_0_	14(45.2)	17(48.6)			11(36.7)	39(67.2)		
N_1_	11(35.5)	15(42.9)			13(43.3)	13(22.4)		
N_2_	6(19.4)	3(8.6)			6(20.0)	6(10.3)		
T downstage, *n* (%)		7(20.0)				23(39.7)		
N downstage, *n* (%)		12(34.3)				35(60.3)		
TRG grade, *n* (%)								
0–1		5(14.3)				12(20.7)		
2–3		11(31.4)				37(63.8)		
NA		19(54.3)				9(15.5)		
Postoperative complications, *n* (%)	9(29.0)	3(8.6)	4.626	**0.031**	7(23.3)	5(8.6)	3.634	**0.050**
obstruction	3	0			5	0		
infection	6	2			2	2		
abdominal hemorrhage	1	1			0	1		
anastomotic leakage	0	0			0	3		
thrombosis	1	0			0	0		

*SEMS* self-expandable metal stents, *NAT* neoadjuvant therapy, *TRG* tumor regression grade

Pathological outcomes revealed no significant differences in tumor size or differentiation between the SEMS-NAT and SEMS groups during both the 2011–2020 and 2021–2025 periods. However, significant differences in postoperative pathological T (pT) and N (pN) stages were observed in the 2021–2025 cohort (*P* = 0.019 and *P* = 0.023, respectively). In the 2021–2025 SEMS-NAT group, 20.7% of patients achieved TRG 0–1, while 63.8% attained TRG 2–3, indicating an improved response to neoadjuvant therapy compared to the 2011–2020 period. Imaging assessments demonstrated T downstaging in 39.7% and N downstaging in 60.3% of cases during 2021–2025, compared to 20.0% and 34.3% in 2011–2020, respectively. 

### Long-term survival of SEMS-NAT group and SEMS group

**Fig. 2:**
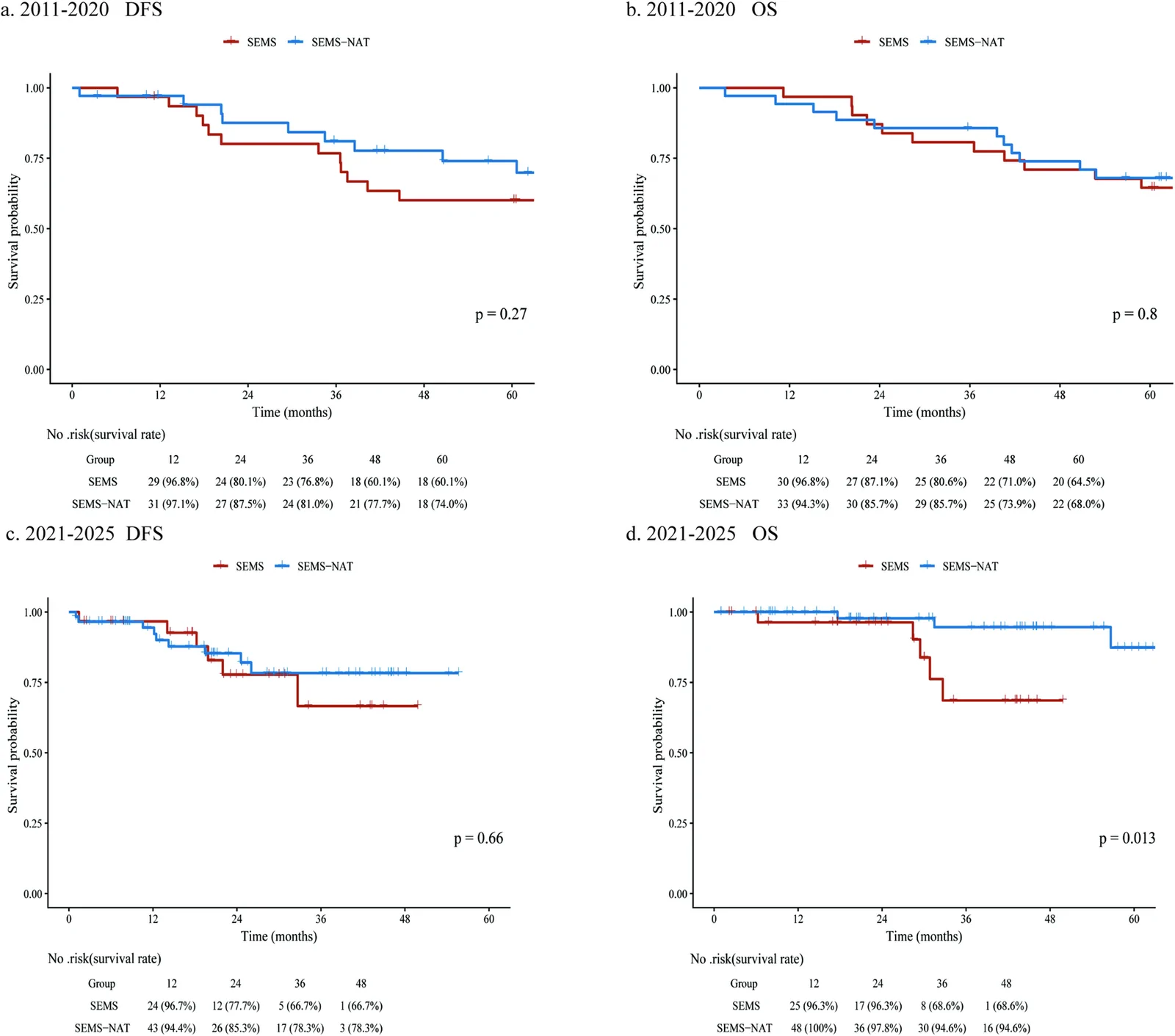
The Kaplan-Meier (KM) curves of disease-free survival (DFS) and overall survival (OS) for SEMS-NAT group and SEMS group were analyzed across the periods 2011–2020 and 2021–2025

This study evaluated survival outcomes in patients with obstructive CRC treated with SEMS-NAT versus SEMS alone during two periods: 2011–2020 and 2021–2025. As illustrated in Fig. 2, the median survival time was not reached in either the SEMS-NAT or SEMS groups within both cohorts. In the 2011–2020 cohort, the median follow-up durations were 65.9 months (range: 3.4 to 133.0) for the SEMS-NAT group and 68.9 months (range: 11.2 to 158.8) for the SEMS group, with 1 patient (3.2%) and 2 patients (5.7%) lost to follow-up, respectively. Although the SEMS-NAT group exhibited marginally higher 3- and 5-year DFS and OS rates, but the KM curves showed no statistically significant differences between groups. In the 2021–2025 cohort, median follow-up times were 41.3 months (range: 1.0 to 68.7) for the SEMS-NAT group and 28.5 months (range: 2.2 to 49.8) for the SEMS group, with no patients lost to follow-up in either group. The SEMS-NAT group demonstrated higher 3-year DFS and OS rates compared to the SEMS group. Notably, neoadjuvant therapy may be an important factor with a statistically significant improvement in OS (*P* = 0.013), whereas no significant benefit was observed in DFS (*P* = 0.66).

### Optimal interval time from SEMS to surgery

**Fig. 3:**
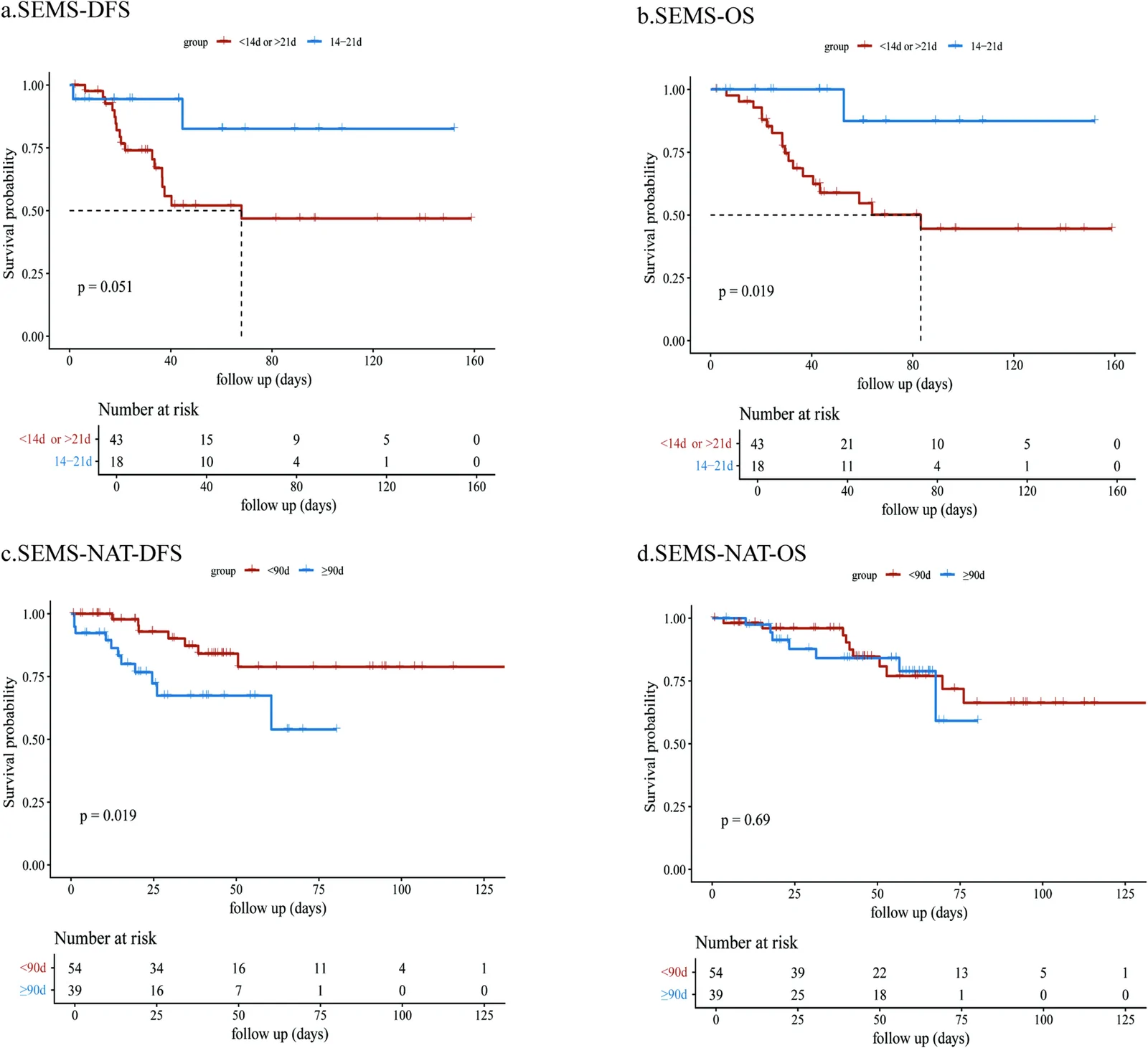
The Kaplan-Meier (KM) curves of disease-free survival (DFS) and overall survival (OS) for different time interval in SEMS-NAT group and SEMS group

This study assessed the optimal timing for surgery by analyzing outcomes in the SEMS-NAT and SEMS groups. Surgical intervals were categorized as follows: SEMS group (≤ 14 days, 14–21 days, ≥ 21 days) and SEMS-NAT group (< 90 days, ≥ 90 days). Short-term outcomes, including surgery type, operative duration, blood loss, length of hospital stay, number of lymph nodes harvested and complication rates, did not differ significantly across the intervals within either group. Detailed results are presented in Table 3. Notably, in the SEMS group, an interval of 14–21 days was associated with improved OS (*P* = 0.019), but the DFS had no significant difference (*P* = 0.051). Conversely, in the SEMS-NAT group, intervals of ≥ 90 days were associated with decreased DFS (*P* = 0.019) but had no significant impact on OS (*P* = 0.69) (Fig. 3). According to these findings, an interval of 14–21 days is recommended for the SEMS group. Whereas for patients receiving neoadjuvant therapy, the interval should not exceed 90 days to avoid impaired survival outcomes.

**Table 3:** Operation outcomes of different interval time in SEMS-NAT group and SEMS group

Variables	SEMS-NAT group			SEMS group			
	< 90 d (*N* = 54)	≥ 90 d (*N* = 39)	*P*	≤ 14 d (*N* = 24)	14–21 d (*N* = 18)	≥ 21 d (*N* = 19)	*P*
laparoscopic surgery, *n* (%)	46(85.2)	32(82.1)	0.685	16(66.7)	14(77.8)	17(89.5)	0.209
Operative time, M (Q1, Q3)	210(177,236)	200(175,260)	0.867	210(180,243.8)	185(145,220)	205(170,255)	0.350
Intraoperative blood loss, M (Q1, Q3)	50(50,100)	50(30,150)	0.899	100(53.8,137.5)	50(27.5,100)	50(50,150)	0.116
Stoma, *n* (%)	4(7.4)	2(5.1)	0.989	7(29.2)	4(22.2)	2(10.5)	0.331
Postoperative hospital, M(Range)	8(3,33)	8(5,33)	0.686	9.0(4.0,30.0)	8.0(4.0,24.0)	8.0(6.0,23.0)	0.217
Lymphnodes removed, M (Q1, Q3)	23.5(19,28)	25(18,33)	0.461	21.0(18.0,26.5)	21.5(15.8,26.5)	21.0(17.0,28.0)	0.968
Positive lymphnodes, M (Q1, Q3)	0(0,1)	0(0,2)	0.551	2.0(0.0,4.8)	0.0(0.0,3.5)	1.0(0.0,2.0)	0.122
Postoperative complications, *n *(%)	8(14.8)	5(12.8)	0.784	7(29.2)	5(27.8)	4(21.1)	0.822

*SEMS* self-expandable metal stents, *NAT* neoadjuvant therapy

## Discussion

Obstructive CRC frequently leads to severe impairment in patients’ physiological functions and quality of life. Although therapeutic advancements such as endoscopic stenting, chemotherapy, and surgical resection have been made, achieving an optimal balance between symptom alleviation and long-term survival remains challenging [25]. In this context, we conducted a multicenter retrospective cohort study involving 154 patients with obstructive CRC who underwent SEMS placement followed by either neoadjuvant therapy or immediate surgical resection. This study aimed to compare the efficacy of these treatment modalities with respect to survival outcomes, postoperative complications, and pathological characteristics. Additionally, we investigated the optimal interval between SEMS insertion and surgery to enhance clinical decision-making. The results offer novel insights into the relationship between preoperative treatment and surgical intervention, underscoring potential strategies to improve patient prognosis and guide personalized therapeutic approaches.

A primary concern regarding the addition of NAT following SEMS has been the potential for increased stent-related complications or delays that could adversely affect surgical outcomes. Our study comprehensively addresses this issue. The comparable and acceptably low rates of stent-related complications observed between the SEMS-NAT and SEMS groups across both time periods indicate that the incorporation of neoadjuvant therapy does not elevate the incidence of stent-related complications, thereby establishing a critical foundation for the combined therapeutic strategy. Consistent findings were reported by Jia Gang Han et al., who observed complication rates of 14.6% vs. 3.9% (*P* = 0.13) [26]. More importantly, the SEMS-NAT group exhibited significantly improved surgical quality. Notably, the intraoperative stoma rate was significantly lower (8.6% vs. 5.2%), with all stomas being prophylactic, and the postoperative complication rate was substantially reduced (8.6% vs. 8.6%), representing some of the most compelling practical benefits. In 2023, Jia Gang Han et al. also demonstrated that the neoadjuvant therapy combined with stenting group had a lower stoma rate compared to the stenting-only group (10.4% vs. 28.8%, *P* = 0.02) [26]. We hypothesize that the observed benefit arises from the synergistic interaction between stent placement and neoadjuvant therapy. Following SEMS placement, mechanical dilation of the obstructed intestinal segment not only alleviates the symptoms associated with acute intestinal obstruction but, more critically, decreases the pressure exerted on the intestinal tract proximal to the anastomosis [27, 28]. In conventional emergency surgery, intestinal wall edema, thickening and compromised blood flow proximal to the obstruction constitute significant risk factors for anastomotic leakage, often necessitating prophylactic stoma creation by surgeons. Conversely, decompression achieved through SEMS placement leads to the resolution of intestinal wall edema and improvement in blood supply, thereby establishing more favorable local tissue conditions conducive to safe primary anastomosis [29, 30]. Additionally, neoadjuvant therapy may mitigate peritumoral inflammation by inducing tumor cell apoptosis and reducing tumor volume, effectively converting stenotic or fixed lesions initially deemed “unsuitable for safe anastomosis” into lesions where “primary anastomosis is feasible” [31–34]. Decompression provides a healthy intestinal wall substrate for anastomosis, while neoadjuvant therapy ensures histological clearance at the resection margins. Patients with obstructive CRC frequently exhibit frailty, a clinical syndrome characterized by diminished multisystem physiological reserves and reduced stress tolerance [35]. A growing body of evidence supports the implementation of frailty assessments as a basis for preoperative risk stratification and perioperative decision-making, with the aim of reducing postoperative complications and mortality [36, 37]. Integrating frailty evaluation into the SEMS-NAT strategy is anticipated to enable more precise identification of high-risk patients in future study [38]. Symptom relief following SEMS placement, combined with the therapeutic window afforded by neoadjuvant treatment, enhances patients’ biological reserves and improves surgical tolerance, thereby establishing optimal conditions for the application of preoperative exercise-based prehabilitation [38]. Collectively, these effects contribute to a reduction in postoperative complications, shortened recovery periods and decreased healthcare costs. A reduction in the ostomy rate not only serves as an indicator of clinical success but also significantly improves patients’ quality of life. A prospective longitudinal observational study published in 2025 demonstrated that, compared to patients without an ostomy, those who underwent the procedure experienced poorer physical (gastrointestinal symptoms), role, emotional, and social functioning at 1 month postoperatively, notably, the difference in physical functioning persisted for up to 12 months [39]. Therefore, for patients with obstructive CRC, the improvement in quality of life associated with avoiding a permanent stoma, including the restoration of bowel function, preservation of body image, and improved psychosocial adaptation, is of considerable importance.

Our analysis of two distinct time cohorts (2011–2020 and 2021–2025) reveals a significant and evolving trend in treatment outcomes. During the 2011–2020 period, although the benefits of surgery were apparent, neither the oncological impact nor the survival advantage of neoadjuvant therapy reached statistical significance. This likely reflects the treatment paradigms of that era, in which neoadjuvant regimens primarily consisted of standard chemotherapy protocols. The therapeutic landscape underwent substantial changes in the 2021–2025 cohort. Specifically, the SEMS-NAT group demonstrated significantly improved postoperative pT and pN stages, a higher proportion of patients achieving major pathological regression (TRG 0–1: 20.7%), and notable rates of imaging-based downstaging. This enhanced tumor response may be partly attributed to multidisciplinary active treatment, high-quality perioperative management, and patient selection criteria, but the updated neoadjuvant treatment regimen is an indispensable key factor [40]. Contemporary regimens demonstrate a more potent effect on both the primary tumor and micrometastatic disease [41, 42], which may be associated with improved OS observed in the SEMS-NAT group in the 2021–2025 period. Of course, the overall improvement in survival time is also closely related to advancements in minimally invasive surgical techniques, the evolution of the tumor ecosystem, and systematic multidisciplinary treatment.

The perspectives of researchers regarding the impact of SEMS implantation on long-term patient survival have developed progressively over time. Previous studies have suggested that SEMS implantation may adversely affect oncological outcomes. Potential mechanisms include: (1) Perforation leading to tumor dissemination: a prospective multicenter cohort study conducted in Japan (208 patients) showed that after standardizing the SEMS implantation procedure, the perforation rate was low (1.9%), but patients who experienced perforation exhibited significantly poorer recurrence-free survival (RFS) (HR = 3.31, 95% CI: 1.03–10.71) [43]. (2) Mechanical manipulation of the tumor: the shearing forces exerted during SEMS placement may induce the release of circulating cell-free DNA and promote invasive pathological features, including nerve invasion and lymphovascular invasion [44]. Moreover, SEMS placement induces adverse gene expression changes in the tumor microenvironment that are associated with tumor progression; these genes are implicated in tumor proliferation, migration, invasion, angiogenesis, and inflammation [45]. In recent years, a growing number of studies have demonstrated that SEMS placement, when performed at specialized centers, is associated with favorable oncological safety. A single-center retrospective study published in 2025 reported no significant differences between the SEMS group and the emergency surgery group regarding overall survival (OS) (*P* = 0.091) and disease-free survival (DFS) (*P* = 0.22) [46]. This indicates that the incidence of complications, specifically perforation, has decreased due to the standardization of SEMS placement techniques and the enhancement of endoscopists’ proficiency, thereby reducing their adverse effects on oncological outcomes. Nevertheless, the influence of SEMS combined with neoadjuvant therapy on long-term survival remains under investigation. A study conducted by the Yang Shi team demonstrated that SEMS combined with neoadjuvant therapy had improved 5-year DFS and OS compared with SEMS group [47]. Two ongoing prospective studies, OUTSTAND and NACSOC-02, are currently in progress [48]. Notably, the NACSOC-02 trial investigates the efficacy of immunotherapy combined with neoadjuvant chemotherapy following stent placement in patients with obstructive left-sided colorectal cancer. This study differs from previous research by incorporating advancements in surgical techniques and alterations in the tumor ecosystem. A stratified analysis was performed on 154 enrolled patients based on the time period. No improvement in DFS or OS was observed in the 2011–2020 cohort, however, an improvement in OS was observed in the 2021–2025 cohort, whereas no benefit was detected for DFS. This discrepancy may be explained by several factors. First, the incidence of stent-related complications, including perforation, was low in both groups, thereby establishing a robust technical foundation for reducing postoperative recurrence in patients. Second, all centers included in this study were large tertiary hospitals, and the endoscopists responsible for stent placement had undergone advanced, standardized training in medical procedures. Last, the follow-up duration for the 2021–2025 cohort remains relatively short, and DFS events may yet emerge.

The interval between SEMS-induced relief of obstruction and elective surgery constitutes a critical window for comprehensive patient optimization. From a metabolic and nutritional standpoint, intestinal obstruction precipitates feeding difficulties, nausea, vomiting, and impaired nutrient absorption, rapidly inducing a catabolic state. SEMS placement restores intestinal continuity, enabling patients to resume oral intake within hours and facilitating physiological nutritional support through oral supplements and enteral nutrition. Empirical evidence indicates that nutritional intervention 7–14 days prior to surgery significantly reduce the incidence of postoperative infectious complications [49, 50]. From an inflammatory and immunological perspective, malignant obstruction elicits a systemic inflammatory response, characterized by elevated inflammatory markers such as C-reactive protein and interleukin-6. Relief of obstruction diminishes intestinal bacterial translocation and attenuates local inflammation, thereby contributing to the restoration of systemic inflammatory homeostasis [51]. Regarding functional reserve and frailty, patients with obstruction frequently experience decreased physical activity due to abdominal distension, pain, and anorexia, resulting in muscle atrophy and reduced functional independence. Frailty has been identified as an independent predictor of adverse outcomes following colorectal cancer surgery [52]. Symptom alleviation subsequent to SEMS placement facilitates the restoration of mobility, thereby enabling preoperative exercise-based prehabilitation interventions, including inspiratory muscle training, endurance training, and resistance training [53]. One of the most practice-changing findings of our study is the identification of the optimal interval between stenting and surgery, which varies fundamentally according to the selected treatment pathway. In 2023, Yu. Tao and colleagues conducted a nationwide cross-sectional survey in China to assess the current management of obstructive left-sided colon cancer. The survey revealed that among hospitals performing stent surgery, 115 hospitals (47.33%) scheduled surgical intervention 1–2 weeks after SEMS placement, while 67 hospitals (27.57%) opted for a 2–3week interval. For patients receiving additional neoadjuvant therapy, 20.63% of hospitals reported an interval of 10–12 weeks between SEMS placement and elective surgery [54]. In this study, the SEMS-alone strategy demonstrated a “therapeutic window” of 14–21 days, associated with superior OS. In stark contrast, the SEMS-NAT pathway requires a prolonged interval to accommodate systemic treatment. Our analysis establishes critical parameters, indicating that intervals exceeding 90 days correlate with poorer DFS. Notably, varying time intervals did not affect short-term surgical outcomes, however, survival outcomes differed significantly. Previous research has similarly reported no significant differences in short-term complications and 5-year long-term survival between early surgery within 8 days and delayed surgery [55]. In 2024, L Ji et al. found that performing radical surgery 14–21 days after SEMS placement improves the rate of minimally invasive procedures and 5-year DFS, but this conclusion is based on data from only 15 patients [56]. In 2024, L. Ji et al. reported that radical surgery performed 14–21 days after SEMS placement improved the rate of minimally invasive procedures and 5-year DFS, but this conclusion was drawn from a limited sample of 15 patients [34]. Additionally, Ji Eun-Na et al. categorized the interval into three groups: <11 days, 11–17 days, and > 17 days. They found that the 11–17-day group experienced the shortest hospital stay and lowest stoma rate [57]. To date, no further studies have identified the optimal timing for surgery in patients receiving neoadjuvant therapy combined with stenting. Notably, in this study, patients with obstructive CRC received 2–3 cycles of NAT during this period. Whether they can achieve similar pathological efficacy as non-obstructive CRC patients remains to be determined. Currently, there is no consensus on the recommended number of NAT cycles for non-obstructive CRC patients. For locally advanced pMMR/MSS colon cancer, based on the FOxTROT and OPTICAL studies, 4–6 cycles are commonly used as a clinical reference range [58, 59]. For locally advanced MSI-H/dMMR colon cancer, a shorter neoadjuvant treatment course (2 cycles) is sufficient [60]. In our study, obstructive CRC patients who received a shorter course of treatment during the time window all achieved tumor downstaging, with MPR (TRG 0–1) of 14.3% and 20.7%, respectively. These figures are numerically lower than the MPR of 18.3% reported in the OPTICAL study (6 cycles of mFOLFOX6 or 4 cycles of CAPOX) and the 27.9% reported by Zhou et al. (4 cycles of FOLFOXIRI) [58, 61]. However, this cohort consisted of obstructive patients who may have had poorer baseline conditions, including increased inflammatory burden and malnutrition secondary to obstruction. The short-course treatment regimen in this study achieved a clinically meaningful balance among treatment duration, tolerability, and efficacy in this special population, providing a feasible balanced approach for obstructive patients who cannot tolerate long-term therapy.

This study presents several notable strengths. A stratified analysis method was employed to account for the impact of technological advancements over the extended enrollment period, thereby enhancing the robustness of the evaluation of surgical outcomes. By aggregating data from three centers, the sample size was significantly larger than that of previous studies. The findings not only confirmed the survival benefits of neoadjuvant therapy combined with stent placement in obstructive CRC but also identified the optimal timing for surgery following stent placement. Nonetheless, the study has several limitations. First, regarding baseline characteristics, propensity score matching should have been used to balance intergroup differences, however, due to the limited sample size, the study relied on demonstrating the absence of statistically significant differences through intergroup comparisons, which may introduce bias. For example, in the 2011–2020 cohort, the preoperative hemoglobin level in the SEMS-NAT group was lower than that in the SEMS group, potentially attributable to declines in serological parameters induced by neoadjuvant therapy. Nevertheless, the hemoglobin levels in the SEMS-NAT group remained within a clinically manageable range and did not adversely affect patients’ nutritional status, rendering it unlikely to have influenced the study outcomes. Second, although efforts were made to include all eligible patients, the limited sample size prevented important subgroup analyses, especially regarding different neoadjuvant regimens, tumor locations, and clinical stages, thus hindering the identification of patient subgroups most likely to benefit. Additionally, TRG data were largely missing in the 2011–2020 cohort, but sensitivity analyses showed no significant impact on the results. Future research with larger sample sizes is warranted to refine these analyses and update the findings. Furthermore, as a retrospective study, it is inherently susceptible to information and selection biases. To minimize information bias, double data entry was employed during the data collection process.

In summary, this study shows that updated neoadjuvant therapy plus stent placement enhances survival in patients with obstructive CRC, underscoring the critical importance of surgical timing. These findings provide valuable insights for the development of future clinical strategies and personalized treatment.

## Supplementary Information

Supplementary Material 1.

## Data Availability

The data were included in the supplement materials. However, the support the findings of this study are available from corresponding author.
