# Supplementary material for: Comparative effectiveness of neoadjuvant therapy combined with stent placement versus stent alone in obstructive colorectal cancer: a multicenter retrospective analysis
Source: BMC Cancer. 2026 Sep 24;26:1139. doi: 10.1186/s12885-026-16728-2 (PMC13602754; doi:10.1186/s12885-026-16728-2)
Supplement: Supplementary file 1 [file 12885_2026_16728_MOESM1_ESM.docx]

Table S1 Sensitivity analysis of pathological evaluation in patients reporting Only tumor regression grade (TRG)

| **Variables** | **2011-2020** | | | | **2021-2025** | | | |
| --- | --- | --- | --- | --- | --- | --- | --- | --- |
|  | **SEMS group(N=31)** | **SEMS-NAT group(N=16)** | **Z/t/χ^2^** | **P** | **SEMS group(N=30)** | **SEMS-NAT group(N=49)** | **Z/t/χ^2^** | **P** |
| **pT stage, n (%)** |  |  | - | 0.340 |  |  | 6.866 | **0.009** |
| T_0-2_ | 0(0.0) | 1(6.2) |  |  | 0(0.0) | 12(24.5) |  |  |
| T_3-4_ | 31(100.0) | 15(93.8) |  |  | 30(100.0) | 37(75.5) |  |  |
| **pN stage, n (%)** |  |  | 4.256 | 0.119 |  |  | 10.461 | **0.005** |
| N_0_ | 14(45.2) | 11(68.8) |  |  | 11(36.7) | 36(73.5) |  |  |
| N_1_ | 11(35.5) | 5(31.2) |  |  | 13(43.3) | 9(18.4) |  |  |
| N_2_ | 6(19.4) | 0(0.0) |  |  | 6(20.0) | 4(8.2) |  |  |
| **T downstage, n (%)*** |  | 1(6.2) |  |  |  | 12(24.5) |  |  |
| **N downstage, n (%)*** |  | 4(25.0) |  |  |  | 20(40.8) |  |  |

**Table S2 Multivariate Cox regression analysis of DFS and OS**

|  | **2011-2020** | | | | **2021-2025** | | | |
| --- | --- | --- | --- | --- | --- | --- | --- | --- |
|  | **HR_DFS_ (95%CI)** | **P** | **HR_OS_ (95%CI)** | **P** | **HR_DFS_ (95%CI)** | **P** | **HR_OS_ (95%CI)** | **P** |
| **Age** |  | 0.366 |  | 0.200 |  | 0.575 |  | 0.787 |
| ≤60 years | Reference |  | Reference |  | Reference |  | Reference |  |
| >60 years | 1.56(0.60,4.07) |  | 1.80(0.73,4.42) |  | 1.46(0.39,5.44) |  | 1.26(0.24,6.62) |  |
| **Gender** |  | 0.955 |  | 0.286 |  | 0.232 |  | 0.549 |
| male | Reference |  | Reference |  | Reference |  | Reference |  |
| female | 0.97(0.38,2.50) |  | 0.61(0.25,1.51) |  | 1.97(0.65,5.95) |  | 1.59(0.35,7.16) |  |
| **Chronic disease history** |  | 0.441 |  | 0.106 |  | 0.156 |  | 0.151 |
| no | Reference |  | Reference |  | Reference |  | Reference |  |
| yes | 0.67(0.25,1.84) |  | 0.43(0.15,1.20) |  | 2.52(0.70,9.04) |  | 3.90(0.61,25.04) |  |
| **Tumor location** |  | 0.971 |  | **0.018** |  | 0.788 |  | 0.891 |
| colon cancaer | Reference |  | Reference |  | Reference |  | Reference |  |
| rectal cancer | 0.96(0.10,10.02) |  | 4.41(1.29,15.08) |  | 1.21(0.31,4.70) |  | 1.15(0.16,8.24) |  |
| **Treament group** |  | 0.296 |  | 0.509 |  | 0.936 |  | **0.044** |
| SEMS | Reference |  | Reference |  | Reference |  | Reference |  |
| SEMS+NAT | 0.62(0.25,1.52) |  | 0.75(0.33,1.75) |  | 0.95(0.29,3.11) |  | 0.13(0.02,0.94) |  |
| **TNM stage** |  |  |  |  |  |  |  |  |
| 0+Ⅰ | **-** |  | **-** |  | Reference |  | Reference |  |
| Ⅱ | Reference |  | Reference |  | 1.84(0.34,10.11) | 0.483 | 5.07(0.37,68.73) | 0.222 |
| Ⅲ | 1.17(0.11,12.97) | 0.898 | 0.50(0.20,1.24) | 0.133 | 0.51(0.14,1.90) | 0.318 | 1.319(0.20,8.59) | 0.772 |
